# Activation of Cx43 Hemichannels Induces the Generation of Ca^2+^ Oscillations in White Adipocytes and Stimulates Lipolysis

**DOI:** 10.3390/ijms22158095

**Published:** 2021-07-28

**Authors:** Egor A. Turovsky, Elena G. Varlamova, Maria V. Turovskaya

**Affiliations:** Federal Research Center “Pushchino Scientific Center for Biological Research of the Russian Academy of Sciences”, Institute of Cell Biophysics of the Russian Academy of Sciences, 142290 Pushchino, Russia; m_turovskaya@mail.ru

**Keywords:** Ca^2+^ oscillations, lipolysis, hemichannels, adipocytes

## Abstract

The aim of the study was to investigate the mechanisms of Ca^2+^ oscillation generation upon activation of connexin-43 and regulation of the lipolysis/lipogenesis balance in white adipocytes through vesicular ATP release. With fluorescence microscopy it was revealed that a decrease in the concentration of extracellular calcium ([Ca^2+^]_ex_) results in two types of Ca^2+^ responses in white adipocytes: Ca^2+^ oscillations and transient Ca^2+^ signals. It was found that activation of the connexin half-channels is involved in the generation of Ca^2+^ oscillations, since the blockers of the connexin hemichannels—carbenoxolone, octanol, proadifen and Gap26—as well as Cx43 gene knockdown led to complete suppression of these signals. The activation of Cx43 in response to the reduction of [Ca^2+^]_ex_ was confirmed by TIRF microscopy. It was shown that in response to the activation of Cx43, ATP-containing vesicles were released from the adipocytes. This process was suppressed by knockdown of the Cx43 gene and by bafilomycin A1, an inhibitor of vacuolar ATPase. At the level of intracellular signaling, the generation of Ca^2+^ oscillations in white adipocytes in response to a decrease in [Ca^2+^]_ex_ occurred due to the mobilization of the Ca^2+^ ions from the thapsigargin-sensitive Ca^2+^ pool of IP_3_R as a result of activation of the purinergic P2Y1 receptors and phosphoinositide signaling pathway. After activation of Cx43 and generation of the Ca^2+^ oscillations, changes in the expression levels of key genes and their encoding proteins involved in the regulation of lipolysis were observed in white adipocytes. This effect was accompanied by a decrease in the number of adipocytes containing lipid droplets, while inhibition or knockdown of Cx43 led to inhibition of lipolysis and accumulation of lipid droplets. In this study, we investigated the mechanism of Ca^2+^ oscillation generation in white adipocytes in response to a decrease in the concentration of Ca^2+^ ions in the external environment and established an interplay between periodic Ca^2+^ modes and the regulation of the lipolysis/lipogenesis balance.

## 1. Introduction

There are two main types of adipose tissue: white and brown White adipose tissue (WAT) stores energy as triglycerides in lipid droplets, whereas brown adipose tissue (BAT) determines adaptive thermogenesis and dissipates energy as heat. Obesity induces a complex remodeling of adipose tissue, which expands to accommodate the excessive caloric intake, with marked changes in its structure and cellular composition, as a result leading to WAT dysfunction [1]. WAT dysfunction is accompanied by excess caloric intake, leading to type 2 diabetes, obesity-driven insulin resistance and an increased susceptibility to many pathophysiological changes, including cardiovascular disease and cancer [2,3,4].

White adipocytes form fat depots in different parts of the body and are the main type of bone marrow cells; their number increases in various pathological conditions, including radiation therapy [5]. However, there is also a third type of adipose tissue cells—“beige” adipocytes—which occurs as inclusions in white fat depots and are characterized by a distinct and different origin and molecular identity from classical brown adipocytes [6,7]. WAT includes different types of cells: adipocytes, pre-adipocytes, macrophages, endothelial cells and mesenchymal stem cells. Up to 50 percent of WAT is adipocytes [8].

Under normal conditions, with a positive caloric balance, free fatty acids (FFA) supplied with blood are stored as triglycerides in mature adipocytes. However, in obesity, “healthy” WAT expansion is achieved by recruiting and differentiating adipose precursor cells [9,10]. It is believed that impairment of these processes and/or remodeling of the extracellular matrix, as well as impaired angiogenesis, can lead to pathological hypertrophy and dysfunction of the adipocytes [11].

Lipolysis stimulated by norepinephrine is provided by the activation of the β-adrenergic receptors, adenylate cyclase and synthesis of cAMP, as well as by the key lipases, hormone sensitive lipase (HSL) and triglyceride lipase (ATGL), and phosphorylation of perilipin. Inhibition of this process is provided by the activation of the α_2_-adrenergic receptors, heterotrimeric Gi protein and αi-subunit, inhibiting adenylate cyclase and cAMP synthesis [12]. The βγ subunits of the Gi proteins also play an important role in the processes of signal transduction from the receptor to the respective targets [13]. Moreover, WAT is an active secretory organ [14], which secretes a number of biologically active substances—adipokines—in response to various stimuli, including stressful ones. The mechanisms of secretion in the cells of most tissues are closely related to changes in the concentration of cytosolic Ca^2+^ ([Ca^2+^]_i_): an increase in its concentration leads to a decrease in the accumulation of triglycerides by white adipocytes, and a decrease, conversely, promotes an increase in body weight [15,16].

The effects of connexins, the activity of which is under the direct control of external Ca^2+^ [17,18], have not been practically investigated for WAT. Global calcium dynamics regulates key intracellular processes in healthy adipose tissue—differentiation, lipid accumulation and insulin signaling [19,20,21]. At the same time, pathological Ca^2+^ signals activate many processes, leading to obesity and other chronic pathological conditions [22]. However, the implication of [Ca^2+^]_i_ in the suppression of lipolysis is also well known and seems to be a complex, poorly studied process [23,24].

White adipocytes, electrically non-excitable cells, are characterized by the formation of connections with the Gap junction channels and their activity is also regulated by changes in the concentration of Ca^2+^ ions [25]. In obesity and type II diabetes, the concentrations of circulating saturated FFA are increased, resulting in the activation of a gap junction that, in turn, leads to the activation of microglia and release of pro-inflammatory factors, ATP and glutamate into the extracellular space. As a result, it may cause neuronal damage and secondary inflammation of glial cells, accompanied by a disturbance of the feeding pattern [25,26,27]. Similar effects of connexin activation can be expected in adipose tissue in the progress of obesity and type II diabetes. However, the effects of hemichannels activation in adipose tissue are still poorly understood.

Vertebrate connexins (Cx) are presented by 20 isoforms of proteins that have four transmembrane domains and differ in the length of the cytoplasmic domain. Cx43 differs from others in its ability to form both connexons and uncoupled “free” Gap junction channels. In addition, Cx43 regulates cellular metabolism, and the gene expression of this connexin affects the expression pattern of other genes [28,29]. Cx43 is also expressed on the inner mitochondrial membrane and its activity is required for the inhibition of MPT megachannels and suppression of the apoptotic process in brain cells [30]. The activity of Cx43 in cardiomyocytes is necessary for the transport of potassium ions from the cytosol [31], the efficient function of complex I and oxidative phosphorylation [32], stimulation of ATP-dependent K^+^-channels and activation of hypoxic preconditioning mechanisms [33].

Connexins play an important role in cell differentiation, adhesion and apoptosis, embryonic development, synaptic transmission, immune responses and carcinogenesis [34]. Connexins are associated with the pathogenesis of types I and II diabetes [35]. In white adipocytes, Cx43 is the main connexin and therefore patients with ODDD are characterized by abnormalities in the physiology of white or brown adipose tissue. It was also found that Cx43 plays a role in adipose-derived stromal cell differentiation into adipocytes [36,37].

Pannexins have a similar structure and function, which are presented by three isoforms in vertebrates [38]. Pannexins can also form channels that open into the extracellular space and their permeability does not depend on the concentration of extracellular Ca^2+^ ions [39], and their ability to form full-fledged intercellular channels remains unconfirmed [40,41]. It is known that pannexins are involved in the mechanism of synaptic ATP release and suppression of the hyperexcitation of neuronal networks through the activation of adenosine receptors on the presynaptic membrane and suppression of glutamate secretion [42]. The role of pannexins and their signaling in adipose tissue is currently poorly understood.

It should be said that an increase in [Ca^2+^]_i_, as a secondary messenger, mediates the action of almost all neurotransmitters and hormones. However, the role of Ca^2+^ ions in the regulation of the balance between lipolysis and lipogenesis in WAT has not been sufficiently studied, including the implication of Ca^2+^ oscillations in the control of both processes. Therefore, it is of interest to study the intracellular mechanism of Ca^2+^ oscillation generation caused by the activation of the Cx43 connexin channels and their impact on the regulation of lipolysis/lipogenesis in white adipocytes.

## 2. Results

### 2.1. Ca^2+^ Responses of White Adipocytes to Ca^2+^-Free Medium Application

**Figure 1:**
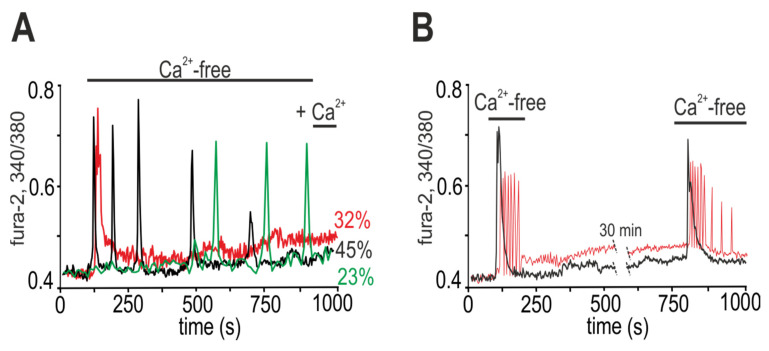
Ca^2+^ signals of white adipocytes due to the application of calcium-free (Ca^2+^-free) medium. (**A**) The Ca^2+^-free medium induces the generation of various Ca^2+^ responses: transient in 32% of adipocytes, Ca^2+^ oscillations without a lag-phase in 45% of adipocytes and Ca^2+^ oscillations with a lag-phase in 23% of adipocytes. Recovery of the extracellular Ca^2+^ concentration to a standard level (+1.2 mM Ca^2+^) does not induce changes in the Ca^2+^ dynamics of adipocytes. Averaged Ca^2+^ signals obtained from 15 adipocytes in one experiment for each curve (*n* = 15) are shown. The experiment was performed in 6 repetitions (*n* = 6) on three separate cell cultures. (**B**) Repeated application of Ca^2+^-free medium to the culture of white adipocytes (after a 30-min period of the cell culture recovery) leads to the generation of Ca^2+^ signals similar in shape and amplitude. The Ca^2+^ signals of single adipocytes are presented.

When the complete medium was replaced with calcium-free medium supplemented with 0.5 mM EGTA (Ca^2+^-free), different Ca^2+^ releases were observed in mature white adipocytes (9 days in vitro, DIV). Directly after stimulation, Ca^2+^ oscillations occurred in 45 ± 11% of cells; Ca^2+^ oscillations were observed in 23 ± 8% of cells after a lag period of 5.2 ± 3.5 min, and transient Ca^2+^ signals were recorded in 32 ± 11% of adipocytes (Figure 1A). Ca^2+^ oscillations in adipocytes occurred without changing the basal level [Ca^2+^]_i_ (Figure 1A, black and green curves), and after the transient signals, a new basal level of [Ca^2+^]_i_ was established (Figure 1A, red curve). When the Ca^2+^-free medium is replaced with a medium containing 1.2 mM Ca^2+^, no changes in [Ca^2+^]_i_ were found in adipocytes (Figure 1A).

The typical calcium signals of the white adipocytes in response to repeated addition of Ca^2+^-free medium are shown in Figure 1B. After the first short-term (~160 s) stimulation of adipocytes by Ca^2+^-free medium, the complete medium (containing 1.2 mM Ca^2+^) was washed, and consequently the Ca^2+^ oscillations were suppressed. To restore the calcium signaling system of the white adipocytes, after a 30-min pause in the registration of [Ca^2+^]_i_, repeated application of the Ca^2+^-free medium was carried out, which led to the release of Ca^2+^ in 87 ± 21% of adipocytes that responded to the first stimulation. Diminution in the number of cells in response to the repeated stimulation was most likely associated with irreversible desensitization of the calcium-transport systems. Moreover, each of the adipocytes was still able to respond to the Ca^2+^-free buffer exclusively in the form of Ca^2+^ responses, which usually occur during the first short-term stimulation; that is, the adipocytes that responded to the first stimulation with an increase in the [Ca^2+^]_i_ impulse responded with the same type of signal to the repeated stimulation (Figure 1B, black curve), but never with oscillations. Such a feature of white adipocytes may be due to an individual set of receptors and expression of calcium-transport systems, which is a good property for inhibitory analysis and establishing the mechanisms underlying the sensitivity of white fat adipocytes to changes in the concentration of extracellular calcium.

Therefore, the replacement of the extracellular medium of the cultured white adipocytes with calcium-free medium caused the generation of two types of Ca^2+^ signals, transient Ca^2+^ responses and Ca^2+^ oscillations, which were suppressed when the concentration of extracellular Ca^2+^ was restored.

### 2.2. Activation of Cx43 Connexin Hemichannels Promotes Generation of Ca^2+^ Oscillations in Response to a Low Extracellular Ca^2+^ Level but This Has No Effect on Ca^2+^ Transients

**Figure 2:**
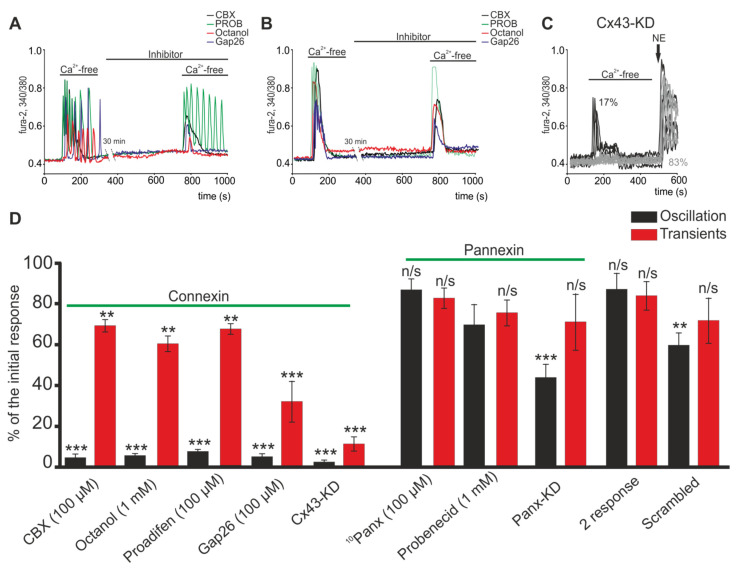
Effect of connexin and pannexin hemichannels blockers on the generation of Ca^2+^ signals in white adipocytes due to the application of Ca^2+^-free medium. (**A**,**B**) Effects of connexin (Carbenoxolone (CBX), 100 µM; Octanol, 1 mM; or Gap26, 100 µM) and pannexin (Probenecid (PROB) 1 mM) hemichannels blockers on the Ca^2+^ oscillation (**A**) and Ca^2+^ transients (**B**) of white adipocytes upon application of Ca^2+^-free medium. Between the first and second application of the Ca^2+^-free medium there was a 30 min pause in the Ca^2+^-dynamics registration (recording). (**C**) Cell knockdown of the Cx43 hemichannels completely suppresses Ca^2+^ oscillations and significantly suppresses the amplitude of Ca^2+^ transients in white adipocytes upon application of a Ca^2+^-free medium. NE—application of 1 µM norepinephrine, an adrenergic receptor agonist. (**D**) Effect of the investigated blockers on the amplitude of the Ca^2+^ oscillations and transients upon repeated Ca^2+^-free application. The amplitude of the Ca^2+^ response to the first application of the Ca^2+^-free medium is taken as 100%. Abbreviations: CBX—Carbenoxolone, 100 µM; Octanol, 1 mM; Proadifen, 100 µM; Gap26, 100 µM; blockers of connexin hemichannels: ^10^Panx, 100 µM and Probenecid, 1 mM; and blockers of pannexin hemichannels, Cx43-KD; Panx-KD—Cx43 and Panx1 knockdown using Gja1 siRNA and Panx1 siRNA; Scrambled—cells transfected with an siRNA whose sequence differed from that of the siRNA against Cx43 and Panx1; 2 response—the amplitude of Ca^2+^ responses to the Ca^2+^-free medium application without inhibitors. The differences between the experimental groups and control are marked with asterisks: ** *p* < 0.01, *** *p* < 0.001; n/s—no significant difference.

It is known that connexin hemichannels can be activated by a decrease in the concentration of extracellular Ca^2+^ ([Ca^2+^]_ex_), and the Cx43 type is most abundantly expressed in white fat adipocytes [36]. Connexin hemichannels blockers, such as carbenoxolone (CBX, 100 µM), octanol (1 mM) or Gap26 (100 µM), an connexin mimetic peptide, completely suppressed Ca^2+^ oscillations in all white adipocytes directly after 30-min preincubation (Figure 2A), and their signals to addition of Ca^2+^-free medium transformed into a single Ca^2+^ transient. Incubation of cells with proadifen (100 µM) (Figure 2D) elicited a similar effect. In the population of white adipocytes responding to the first addition with transient calcium signals, the hemichannels blockers did not affect the generation of calcium signals in response to repeated addition of the Ca^2+^-free medium (Figure 2B). In general, no decreases in the amplitude of the Ca^2+^ transients were found (Figure 2D). Like the connexin hemichannels, pannexin 1 hemichannels (Pannexin-1) are also expressed in white adipocytes and perform several important physiological functions [43]. Incubation of white adipocytes with a pannexin blocker, probenecid (PROB, 1 mM), did not affect the generation of Ca^2+^ oscillations (Figure 2A) and the amplitude of the Ca^2+^ transients (Figure 2D). After addition of Ca^2+^-free medium, the highly selective peptide blocker Pannexin-1 (^10^Panx, 100 µM) also did not affect the Ca^2+^ signals of the white adipocytes (Figure 2D). Knockdown of the Panx-1 gene can lead to a decrease in the number of cells with Ca^2+^ oscillations without significantly changing the number of adipocytes with Ca^2+^ transients (Figure 2D, Panx-KD).

Interestingly, Cx43 gene knockdown using Gja1 siRNA completely inhibits Ca^2+^-free medium-induced Ca^2+^ oscillations (Figure 2C), but also caused a statistically significant decrease in the amplitude of Ca^2+^ transients (Figure 2D). Herewith, cellular Cx43 knockdown does not change the density of a cell culture, cell morphology (not shown) nor their response to physiological stimuli (Figure 2C—NE). The response of white adipocytes was a high-amplitude increase in [Ca^2+^]_i_ after application of 1 µM of noradrenaline, which is typical for normal cell cultures of mature white adipocytes [44].

**Figure 3:**
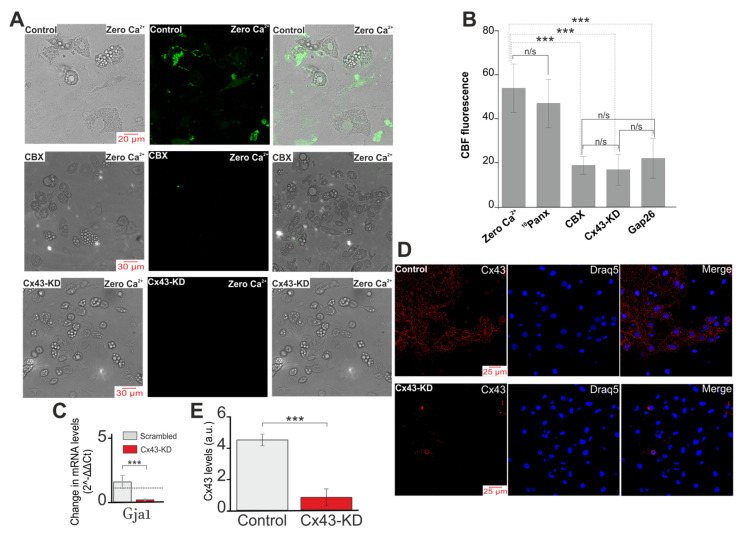
Opening of connexin hemichannels in response to Ca^2+^-free medium (Zero Ca^2+^). (**A**,**B**) Representative images (**A**) and summary data (**B**) of CBF fluorescence in cultured white adipocytes illustrating background dye loading (Control) and intracellular CBF accumulation (loading) in response to Ca^2+^-free medium (Control + Zero Ca^2+^) in the absence of inhibitors and presence of CBX (100 µM), ^10^Panx (100 µM) and Gap26 (Mimetic peptide, Gap26, 100 µM, an Cx43-blocking peptide) and conditions of Cx43 gene knockdown using Gja1 siRNA (Cx43-KD). The efficacy of Cx43-KD was tested using real-time PCR (**C**) and immunocytochemistry staining with antibodies against Cx43 (**D**). (**E**) Influence of Cx43 gene knockdown on the expression level of Cx43. Qualitative data are presented on the expression of Cx43 in the form of the average Cx43 intensity values in the summary bar charts, representing the mean ± SEM from 170 cells for each column. The differences between the experimental groups are marked with asterisks: *** *p* < 0.001, n/s—no significant difference.

It is known that connexin hemichannels in an opened state are permeable to carboxyfluorescein [45]. In the presence of carboxyfluorescein in the incubation media, application of Ca^2+^-free medium to white adipocytes facilitated intracellular accumulation of the dye (Figure 3A,B), which may indicate the opening of membrane channels. The accumulation of the dye caused by stimulation of the Ca^2+^-free medium was prevented by CBX (Figure 3A,B) or the peptide blocker Cx43—Gap26 (Figure 3B) and also the knockdown of the Cx43 gene (Figure 3A,B). Pre-incubation of white adipocytes with the blocker of Pannexin-1—^10^Panx (100 µM) did not affect intracellular accumulation of the dye (Figure 3B), which confirms that pannexins are not involved in the response of white adipocytes to a decrease in the concentration of external calcium.

Thus, a decrease in the concentration of extracellular Ca^2+^ caused the generation of Ca^2+^ oscillations in white adipocytes due to the activation and opening of connexin, mainly Cx43, but not pannexin hemichannels. However, the blockers of connexin and pannexin hemichannels did not affect the generation and amplitude of Ca^2+^ transients, which was probably due to the activation of other signaling pathways.

### 2.3. Decrease in Extracellular Ca^2+^ Ions Induces Vesicular ATP Release by White Adipocytes

**Figure 4:**
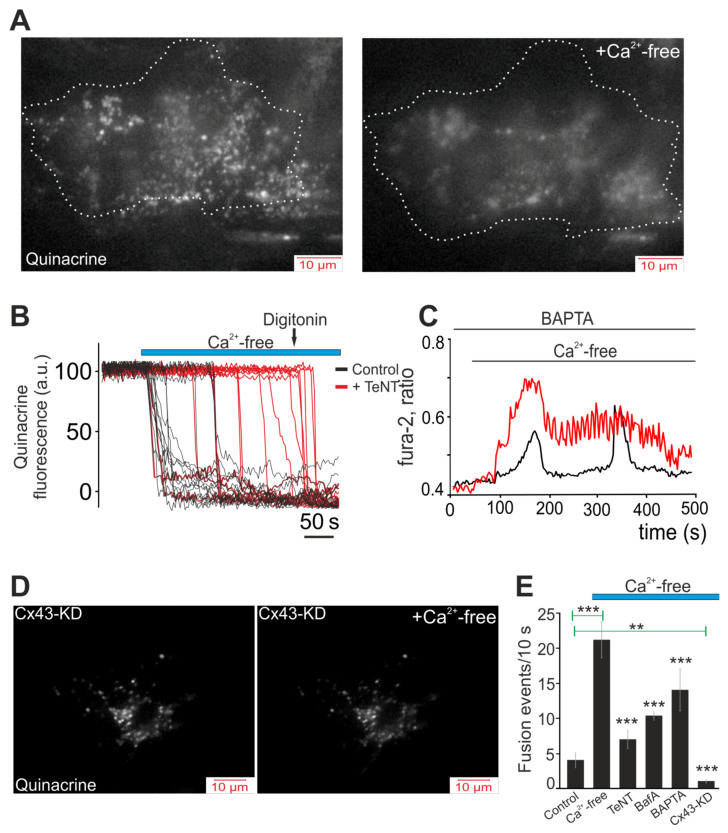
Vesicular ATP release by white adipocytes in response to Ca^2+^-free medium application. Effects of secretion inhibitors, knockdown of Cx43 and Ca^2+^ chelators on the vesicular secretion. (**A**,**D**) Images of the near-membrane localization of ATP-containing vesicles stained with quinacrine, obtained using TIRF microscopy before and after application of the Ca^2+^-free medium (+Ca^2+^-free), to control white adipocytes (**A**) and cells with Cx43 knockdown (Cx43-KD) (**D**). A single white adipocyte is presented. (**B**) Dynamics of ATP-containing vesicle secretion obtained using TIRF microscopy, reflecting increased secretion (decrease in quinacrine fluorescence) upon application of the Ca^2+^-free medium in the control (black curves) and with 50 ng/mL TeNT (red curves), an inhibitor of Ca^2+^-dependent vesicular fusion. (**C**) Effect of pre-incubation of white adipocytes for 40 min with 50 µM of the Ca^2+^ chelator, BAPTA-AM, on the Ca^2+^-free medium-induced Ca^2+^ oscillations of white adipocytes. Shown is the typical Ca^2+^ responses of the white adipocytes. Shown is the typical Ca^2+^ responses of 27 ± 11% (red line) and 32 ± 8% (black line) white adipocytes. (**E**) Summary data illustrating the peak frequency of the Ca^2+^-free medium-induced fusion of the ATP-containing vesicles recorded in white adipocytes without stimuli (Control), with Ca^2+^-free medium application and the Ca^2+^-free medium with 50 ng/mL tetanus toxin (TeNT), an inhibitor of Ca^2+^-dependent vesicular fusion, 1 µM Bafilomycin A1 (BafA), a vacuolar ATPase inhibitor, 50 µM BAPTA-AM (BAPTA), a Ca^2+^ chelator, and Cx43-KD—Cx43 gene knockdown using Gja1 siRNA. Statistical analyses were performed by paired *t*-test. Significance between groups means: ** *p* < 0.01 and *** *p* < 0.001.

A fairly large range of active molecules, including ATP, can be secreted through hemichannels [46]. Quinacrine, a derivative of acridine, is a weak base that binds ATP with high affinity [47]. The cells were loaded with the fluorescent probe quinacrine to visualize the ATP-containing vesicles and to research the dynamics of their secretion in white adipocytes during a decrease in [Ca^2+^]_ex_ and opening of Cx43. Figure 4A shows a single white adipocyte loaded with quinacrine prior to stimulation with Ca^2+^-free medium, and the image obtained by TIRF-microscopy shows a great number of ATP-containing vesicles. After a 5-min exposure to Ca^2+^-free medium, almost complete vesicular ATP release was observed (Figure 4A, Ca^2+^-free). Time analysis of the dynamics of the secretion showed that the secretion of most ATP-containing vesicles occurred within the first 20–30 s after addition of the Ca^2+^-free medium (Figure 4B). It was shown that in other non-excitable cells—for example, astrocytes—ATP secretion is a Ca^2+^-dependent process [48] and incubation with a tetanus toxin (TeNT, 50 ng/mL), an inhibitor of Ca^2+^-dependent vesicular fusion, led to the development of a lag phase followed by vesicular ATP release after addition of the Ca^2+^-free medium (Figure 4B, +TeNT). Furthermore, the number of secreted vesicles usually decreased (Figure 4E). This experiment showed that Ca^2+^ ions are needed for vesicular ATP release, and taking into account that after addition of the Ca^2+^-free medium, the Ca^2+^ ions were absent in the external environment; then, probably, the adipocytes used the accumulated Ca^2+^. In fact, incubation of white adipocytes with a Ca^2+^ chelator, BAPTA-AM, for an hour changed the form of the Ca^2+^-free medium-induced Ca^2+^ oscillations. As a result, either the interval between oscillations increased (Figure 4C, black curve), or the amplitude of the Ca^2+^ oscillations decreased and their frequencies increased (Figure 4C, red curve). Additionally, at the level of vesicular ATP release, incubation with BAPTA-AM, in general, significantly reduced the number of secreted vesicles (Figure 4E). A decrease in the number of secreted vesicles in Ca^2+^-free medium was also observed upon incubation with Bafilomycin A1 (BafA), a vacuolar ATPase inhibitor (Figure 4E), or cellular knockdown of Cx43, when ATP secretion was completely suppressed in white adipocytes (Figure 4D,E).

As a result, activation of the Cx43 hemichannels occurred in response to a decrease in [Ca^2+^]_ex_, thereby leading to vesicular ATP release, a process dependent on the intracellular concentration of the Ca^2+^ ions.

### 2.4. Activation of Cx43 in Response to a Decrease in [Ca^2+^]_ex_ Contributes to Phosphoinositide Signaling Pathway and Activation of G-proteins

**Figure 5:**
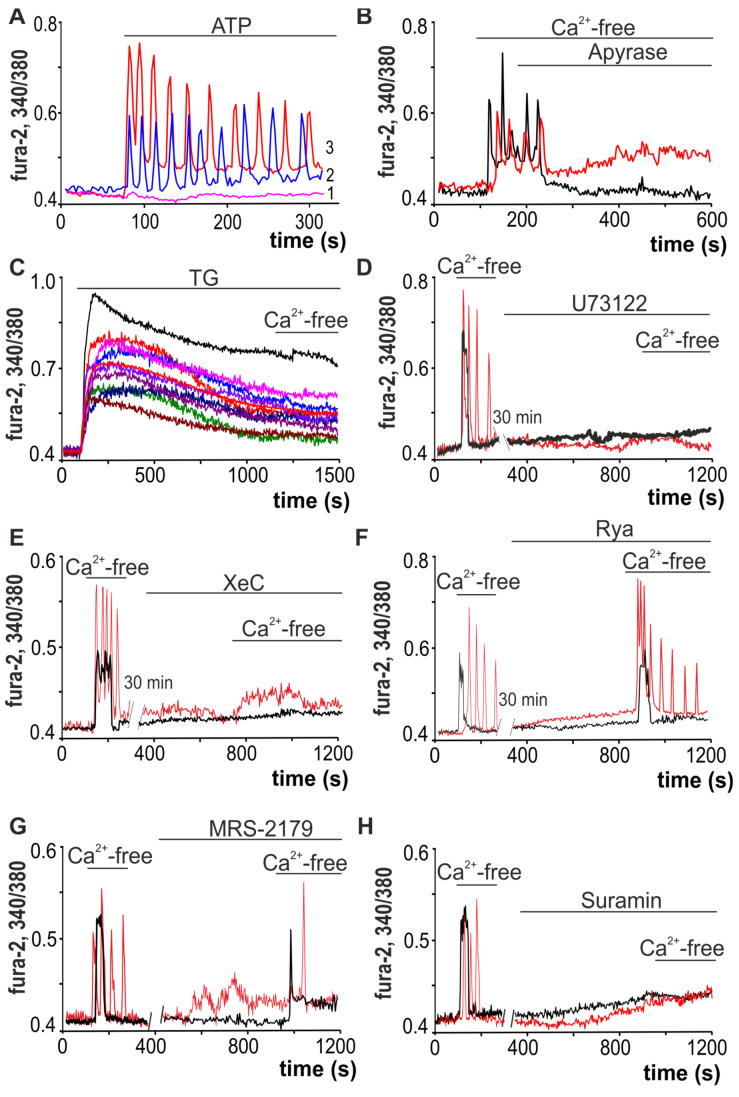
Mechanisms underlying the Ca^2+^ responses of white adipocytes to decreases in external Ca^2+^. (**A**) Application of 10 µM ATP induces the generation of Ca^2+^ oscillations without changing the baseline [Ca^2+^]_i_ level in 22 ± 16% of adipocytes (2) and with an increased baseline of [Ca^2+^]_i_ level in 47 ± 11% of adipocytes (3), while in 31 ± 11% of adipocytes Ca^2+^ signals are absent. (**B**) Application of apyrase (apyrase, 35 units/mL), an enzyme that hydrolyzes ATP, against the background of Ca^2+^-free medium-induced Ca^2+^ oscillations, leads to their rapid and complete inhibition. (**C**) Ca^2+^-free medium-induced Ca^2+^ rises are prevented by discharge of the TG-dependent stores with 10 µM thapsigargin (TG). (**D**–**F**) Ca^2+^ signals due to the application of the Ca^2+^-free medium are completely suppressed by PLC (U73122, 10 µM, (**D**)) and IP_3_R (XeC, Xestospongin C, 1 µM, (**E**)) inhibitors and do not depend on RyR inhibition (Rya, Ryanodine, 10 µM, (**F**)). (**G**) Ca^2+^-free medium-induced Ca^2+^ oscillations of white adipocytes are suppressed by the P2Y1-receptor antagonist—MRS-2179 (30 µM)—but the Ca^2+^ signals have transient shapes. (**H**) Ca^2+^ signals of the white adipocytes after application of Ca^2+^-free medium are completely suppressed in the presence of suramin, an uncoupler of G-proteins and an antagonist of the P2X and P2Y receptors.

Application of ATP (10 µM) to white adipocytes promoted the generation of predominantly Ca^2+^ oscillations that occur without any change in the basal level of [Ca^2+^]_i_ in 22 ± 16% of adipocytes (Figure 5A, designation 2), or with an increase in the basal level of [Ca^2+^]_i_ in 47 ± 11% of adipocytes (Figure 5A, designation 3). Ca^2+^-free medium-induced Ca^2+^ oscillations were rapidly suppressed with application of apyrase (apyrase, 35 U/mL, Figure 5B), an ATP-hydrolyzing enzyme. At the same time, in one population of white adipocytes, the basal [Ca^2+^]_i_ level returned to that observed at rest, while in another population no similar effect was found.

To generate Ca^2+^ signals, cells can use both the input of Ca^2+^ ions from outside and mobilization from intracellular calcium stores. Depletion of calcium from the endoplasmic reticulum (ER) of the cells incubated with thapsigargin (TG, 10 µM), the SERCA inhibitor, led to disappearance of both Ca^2+^ oscillations and Ca^2+^ transients in white adipocytes after a decrease in [Ca^2+^]_ex_ (Figure 5C). U73122 (10 µM)*,* a phospholipase C (*PLC*) inhibitor, completely inhibited Ca^2+^ release from white adipocytes after repeated addition of Ca^2+^-free buffer (Figure 5D). Similarly, inhibition of the IP_3_R by Xestospongin C (XeC, 1 µM, Figure 5E) prevented the generation of Ca^2+^ oscillations and transients in all adipocytes in response to the first addition of Ca^2+^-free buffer. However, treatment of cells with ryanodine (Rya, 10 μM, Figure 5F), an inhibitor of ryanodine receptors, did not affect the generation of Ca^2+^ signals in adipocytes.

MRS-2179 (30 µM, Figure 5G), the P2Y1 receptor antagonist, completely suppressed the Ca^2+^ oscillations of white adipocytes, but the signals in response to addition of Ca^2+^-free medium were in the form of single fast Ca^2+^ impulses. Furthermore, suramin (5 µM, Figure 5H), an uncoupler of G-proteins [49], completely inhibited the Ca^2+^ release from adipocytes, which suggested that G-proteins are involved in the activation of Ca^2+^ transients in response to a decrease in [Ca^2+^]_ex_; this issue requires further study.

Thus, the signaling pathway involved in generation of Ca^2+^ oscillations by white adipocytes in response to a decrease in extracellular Ca^2+^ includes mobilization of Ca^2+^ ions from the thapsigargin-sensitive Ca^2+^ pool of endoplasmic reticulum with participation of phospholipase C and IP_3_R activation. In this case, paracrine activation of P2Y1 takes place in response to the opening of the Cx43 hemichannels and vesicular ATP release by adipocytes, which responded to addition of the Ca^2+^-free medium that leads to local signal propagation throughout the cells of WAT.

### 2.5. A Decrease in [Ca^2+^]_ex_ Stimulates Lipolysis and Correlates with Generation of Ca^2+^ Oscillations in White Adipocytes

**Figure 6:**
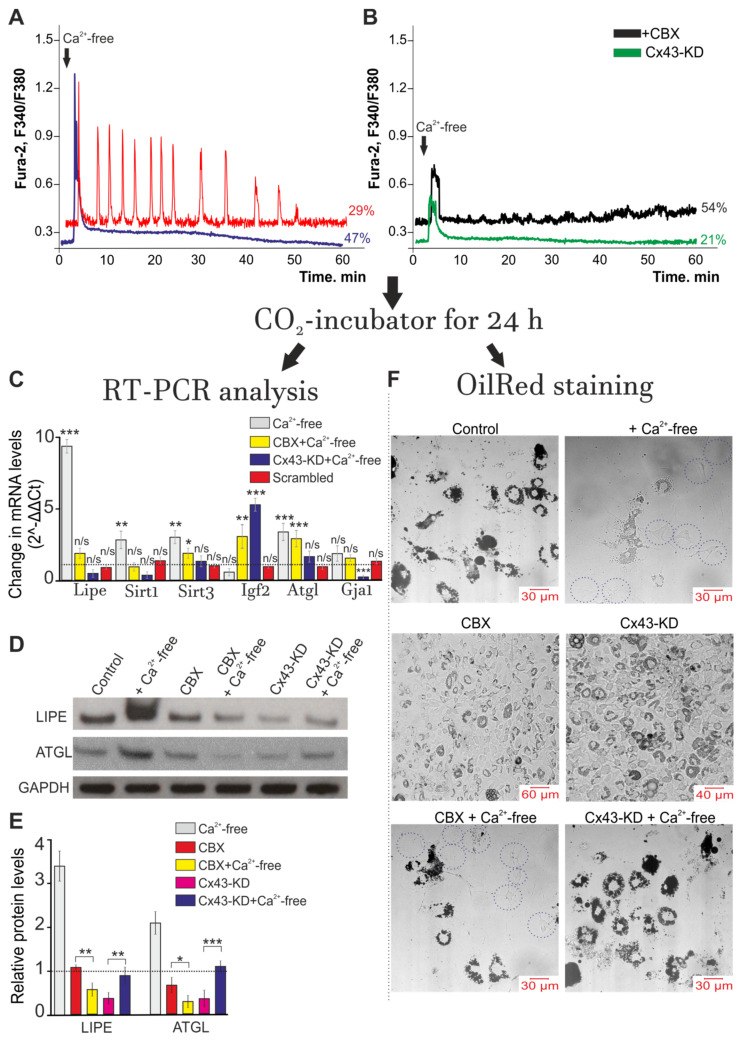
Ca^2+^ oscillations induced by Ca^2+^-free medium application activate the lipolysis process and correlate with changes in the expression of key genes in white adipose tissue. (**A**,**B**) Control (**A**) white adipocytes and cells with Cx43 gene knockdown using Gja1 siRNA (Cx43-KD, (**B**)) were exposed to Ca^2+^-free medium for 1 h and then returned to a CO_2_ incubator for 24 h. Red line—Ca^2+^ oscillations in 29 ± 14% and Ca^2+^ transients (blue curve) in the 47 ± 9% of white adipocytes. In Panel (**B**)—black curve (+CBX): Ca^2+^-free medium application was performed with 100 µM CBX. (**C**) Effects of the Ca^2+^-free medium on the expression of genes in white adipocytes + CBX and *Cx43* gene knockdown cells (Cx43-KD) after 24 h. All values are given as the mean ± SEM. Scrambled—cultures transfected with an siRNA whose sequence differed from that of the siRNA against Cx43. Gene expression level was normalized to the reference gene *Gapdh* and was presented relative to the control (adipocytes were not exposed to Ca^2+^-free medium), which was considered as 1 (dashed line). A comparison of the experimental groups with the control was made. The differences between the experimental groups and control are marked with asterisks: * *p* < 0.05, ** *p* < 0.01, and *** *p* < 0.001. n/s—no significant difference. (**D**) The protein abundance of LIPE and ATGL in control, Cx43-KD, CBX-treated, Ca^2+^-free-treated, CBX + Ca^2+^-free-treated and CX43-KD + Ca^2+^-free-treated white adipocytes. The data were obtained by Western blotting. (**E**) Relative protein levels of LIPE and ATGL in the experimental groups from Panel (**D**). The data were presented as the mean ± SD of at least three independent experiments. Glyceraldehyde–3–phosphate dehydrogenase (GAPDH) was used as an internal control for normalization. The differences between the experimental groups are marked with asterisks: * *p* < 0.05, ** *p* < 0.01, and *** *p* < 0.001. (**F**) Effect of the Ca^2+^-free medium on the accumulation of lipid droplets in control white adipocytes (Control, without Ca^2+^-free), CBX-treated cells (CBX) after 1 h of Ca^2+^-free medium exposure in the control (+Ca^2+^-free) and the presence of 100 µM CBX (CBX + Ca^2+^-free), as well as in Cx43 gene knockdown cells (Cx43-KD) and Cx43-KD with Ca^2+^-free medium exposure (Cx43-KD + Ca^2+^-free).

It is known that Ca^2+^ oscillations can regulate many physiological processes both in excitable and electrically unexcitable tissues [50]. Replacement of the extracellular medium with a calcium-free medium resulted in generation of Ca^2+^ oscillations, on average, in 29 ± 14% of cells, which lasted for 52 ± 5 min and tended to decrease the amplitude of the Ca^2+^ signals (Figure 6A, red curve). At the same time, 47 ± 9% of the white adipocytes showed transient Ca^2+^ signals in the presence of the Ca^2+^-free solution and no Ca^2+^ oscillations occurred in this cell population for 60 min of recording, and the increased basal [Ca^2+^]_i_ level returned to the basal level observed at resting by the end of the cell response during the experiment (Figure 6A, blue curve). The use of a Ca^2+^-free solution and application of 100 µM of CBX (Figure 6B, black curve) or Cx43 knockout (Cx43-KD, Figure 6B, green curve) to adipocytes generated Ca^2+^ transients exclusively in 54 ± 18% and 21 ± 7% of cells, respectively, the amplitudes of which were usually lower as compared to the control (Figure 6A, blue curve). After registration of the Ca^2+^ dynamics of the adipocytes after adding a Ca^2+^-free solution, the cell cultures were put into the CO_2_ incubator again for 24 h. Then, total RNA was isolated from one part of the cells and used for PCR analysis, while the other part of the cells was fixed and loaded with a probe that stained lipid inclusions (OilRed, Figure 6D). The expression of *Gja1*, a gene encoding Cx43, did not significantly change after using the Ca^2+^-free solution in combination with the connexin blocker CBX (Figure 6A), while the cell knockdown of *Gja1* resulted in almost complete suppression of expression of this gene. After incubation of the white adipocytes with a Ca^2+^-free solution during 24 h, an increase in the level of expression of the genes *Lipe*, *Sirt1*, *Sirt3* and *Atgl*, encoding hormone-sensitive lipase, sirtuins 1 and 3 and triglyceride lipase, of 8.6, 3.1, 3.3 and 3.6 times, respectively, was observed (Figure 6C). Simultaneously, the expression of the Igf2 gene encoding insulin-like growth factor-2 was suppressed by 54% (Figure 6C). The use of Scrambled-siRNA did not significantly change the gene expression.

Western blotting showed that after incubation of white adipocytes with a Ca^2+^-free solution during 24 h, the LIPE and ATGL protein content (Figure 6D) increased by 3.5 and 2.3 times (Figure 6E) compared to the control, which is in good agreement with the PCR analysis (Figure 6C). Incubation of cells with 100 µM CBX did not lead to a significant change in the abundance of LIPE and ATGL protein levels, but application of Ca^2+^-free medium with CBX led to a decrease in the level of both these proteins in comparison with the control, which was incubated in a Ca^2+^-containing medium. Cx43-KD also led to a significant decrease in LIPE and ATGL in white fat cells, and incubation of these cell cultures in Ca^2+^-free medium resulted in a slight increase in the levels of these proteins (Figure 6D,E).

Data on changes in gene expression coincide with those on the decrease in the number of adipocytes loaded with lipid droplets (Figure 6F, +Ca^2+^-free), compared with the control (Figure 6F, Control) (adipocytes on the 9 DIV, without Ca^2+^-free); this may indicate an activation of lipolysis. The addition of a Ca^2+^-free solution for 60 min in the presence of CBX (100 µM) blocker led to a significant increase in the expression level of 2 out of 5 studied genes, such as *Igf2* (by 3.2 times) and *Atgl* (by 2.9 times) (Figure 6C). Incubation of cells with CBX reduced the number of adipocytes filled with lipid inclusions (Figure 6F, CBX). The use of a Ca^2+^-free solution in combination with CBX resulted in less morphological changes in the white adipocytes (Figure 6F, CBX + Ca^2+^-free) compared to those observed when only the Ca^2+^-free solution was used: the number of adipocytes loaded with lipid droplets decreased compared to the control (Figure 6F, Control), but increased when the Ca^2+^-free solution was added (Figure 6D, +Ca^2+^-free). A knockdown of the *Gja1* gene suppressed Ca^2+^ oscillations in white adipocytes in response to application of a Ca^2+^-free medium and promoted an increase of lipid droplets in white adipocytes (Figure 6F, Cx43-KD). Application of the Ca^2+^-free medium to the Cx43-KD cell cultures suppressed the expression of the *Lipe* (by 19%) and *Sirt1* (by 31%) genes but induced a 5.8-fold increase in *Igf2* expression (Figure 6C); this correlated with the elimination of the effect the Ca^2+^-free medium had on the intracellular accumulation of the lipid droplets (Figure 6F, Cx43-KD + Ca^2+^-free). The culture of adipocytes stained with OilRed was morphologically similar to the control (Figure 6F, control).

**Figure 7:**
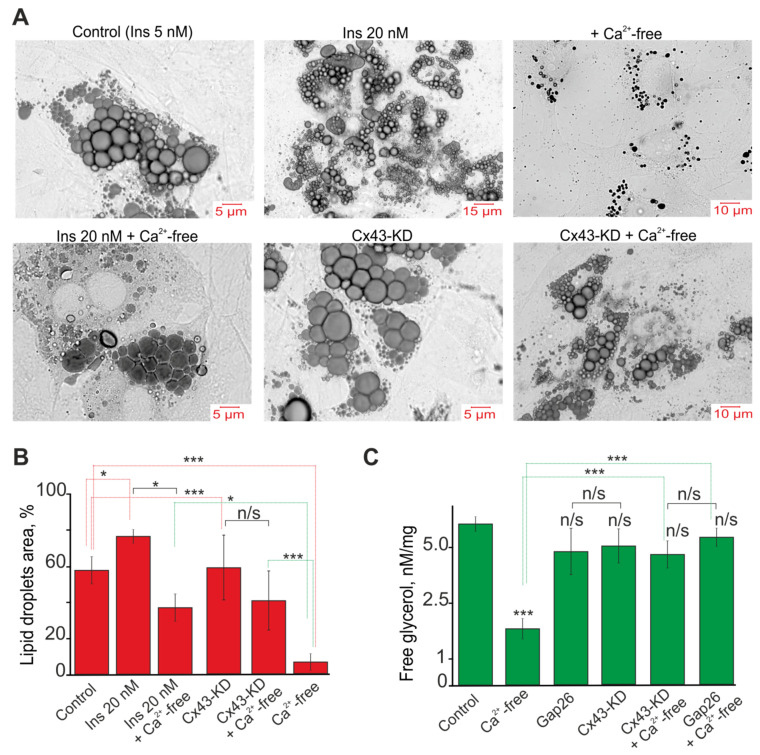
Effect of Ca^2+^-free medium on the accumulation of lipid droplets (**A**), their area (**B**) and free glycerol (**C**). (**A**) Lipid droplets in the white adipocytes grown with the addition of 5 nM (Control) and 20 nM insulin from the first day of cultivation and the effect on them 24 h after 60 min of incubation with Ca^2+^-free medium (+Ca^2+^-free and Ins 20 nM + Ca^2+^-free). The accumulation of lipid droplets in adipocytes with Cx43-KD and in them after Ca^2+^-free medium application is designated Cx43-KD and Cx43-KD + Ca^2+^-free, respectively. The cells are stained with the Oil Red probe. (**B**) The area of lipid droplets in white adipocytes relative to the area of the cells themselves. The area of adipocytes is taken as 100%. Mean values ± SE are presented. For each column, 150 white adipocytes were analyzed. (**C**) Concentration of free glycerol in the culture of white adipocytes in the control, after 24 h of incubation with 100 μM Gap26, a blocker Cx43, and 24 h after 60 min of incubation with the Ca^2+^-free medium against the background of Gap26 and cells with Cx43-KD. A 200 µg white fat cell lysate was used for measurement. The differences between the experimental groups are marked with asterisks: * *p* < 0.05 and *** *p* < 0.001, n/s—no significant difference.

White mouse adipocytes in culture are characterized by the presence of a large number of lipid droplets of various sizes (Figure 7A). To reveal the differences in the effects of the cultivation conditions, Cx43-KD and a Ca^2+^-free medium, we used a method for assessing the area of lipid droplets in relation to the area of cells. For this, all lipid droplets were isolated in each adipocyte using ImageJ and the Analyze Particles Plugin and their areas were determined. The area of the cell itself was also measured. In the control, the area of the lipid droplets on average occupied 60 ± 11% of the area of the entire cell (Figure 7A,B, Control), and the cultivation of adipocytes with a high insulin concentration in the medium led to an increase in this indicator to 76 ± 7% (Figure 7A,B, Ins 20 nM). Cx43-KD did not significantly affect the area of the lipid droplets (Figure 7A,B, Cx43-KD). Incubation of the cells, which was grown with 20 nM insulin and Cx43-KD cells for 60 min in Ca^2+^-free medium after 24 h, led to a decrease in the area of lipid droplets to 38 ± 11% (Figure 7A,B, Ins 20 nM + Ca^2+^-free) and 41 ± 20% (Figure 7A,B, Cx43-KD + Ca^2+^-free), respectively. In turn, the incubation of control adipocytes with Ca^2+^-free medium led to a decrease in the cell area to 5 ± 3% (Figure 7A,B, + Ca^2+^-free), and the lipid droplets themselves became significantly smaller in size.

Measurements of the free glycerol in the lysate of the white adipocytes after the experiments were performed, according to the scheme in Figure 6, show that incubation of cells for 60 min in Ca^2+^-free medium after 24 h reduces the concentration of glycerol by 68 ± 7%, compared with the control (Figure 7C). At the same time, incubation of Cx43-KD and adipocytes in Ca^2+^-free medium after inhibition of Cx43 by Gap26 does not significantly affect the glycerol concentration (Figure 7C). Cx43 itself, as well as knockdown or incubation of cells with Gap26 also did not affect the glycerol concentration (Figure 7C).

**Figure 8:**
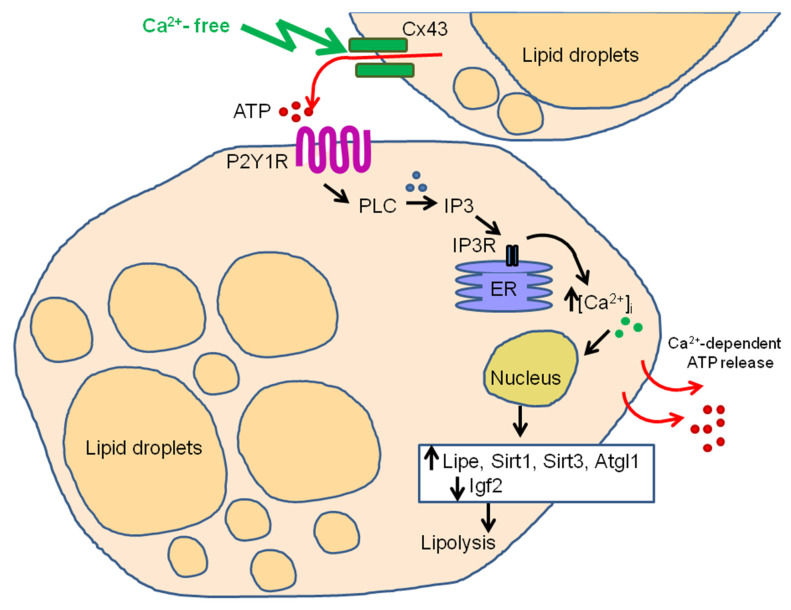
Hypothesized model of Ca^2+^-free medium-induced Ca^2+^ signaling correlated with the activation of lipolysis. Ca^2+^ -free medium activates Cx43 hemichannels and induces vesicular ATP release. ATP release activates of the P2Y1 purinoreceptors of nearby adipocytes and triggers a signaling cascade involving the phosphoinositide signaling system—phospholipase C (PLC)—which leads to the mobilization of Ca^2+^ ions through the IP_3_ receptor (IP_3_R) from the endoplasmic reticulum (ER). The increase in [Ca^2+^]_i_ in white adipocytes is periodic in the form of prolonged Ca^2+^ oscillations in the cytosol, which causes Ca^2+^-dependent ATP release and paracrine activation of the fat cells’ monolayer. This behavior of the Ca^2+^ signaling system correlates further with an increase in the expression of the key lipolysis genes *Lipe*, *Sirt1* and *Atgl1*, and a decrease in the expression of the *Igf2* gene. As a result, there is a decrease in the number of lipid droplets and the level of glycerol.

Thus, we observed a change in the expression of key genes and their proteins involved in the regulation of lipolysis 24 h after activation of Cx43 and generation of Ca^2+^ oscillations in white adipocytes, which was accompanied by a diminution in the number of adipocytes loaded with lipid droplets. It should be noted that inhibition or Cx43 knockdown not only suppressed Ca^2+^ oscillations but also tended to decrease the level of genes encoding the key enzymes of lipolysis (Figure 8).

## 3. Discussion

The physiological effects of connexins depend on both the type of connexin hemichannel and the type of tissue in which it is expressed, and, probably, the type of exposure. For example, neointima formation following balloon catheter injury is significantly reduced in heterozygous Cx43 knockout mice, suggesting a correlation between neointima formation and high levels of Cx43 during the inflammatory response to injury [51]; i.e., participation of this hemichannel in the inflammatory response.

It has been found that disturbances in brain energy metabolism during hypoxia/ischemia lead to increased expression of Cx43 and inhibition of this mechanism can be destructive to cells [52,53], most likely through a disconnected astrocytic network. Reoxygenation after ischemia also leads to a change in the level of phosphorylation and expression of Cx43 in astrocytes. [54]. On the one hand, connexins blockers exert a neuroprotective effect during brain ischemia and [55], on the other hand, Cx43 activity may contribute to the development of the effect of hypoxic preconditioning [56]. Cx43 is highly expressed both in the mesenchymal fraction of the stem cells and in resident adipose-derived stem cells. The expression level of this connexin is increased during differentiation of adipocytes [36,57], distinguishing them significantly from the astrocytes, suggesting other physiological functions of the connexin. Thus, our work established a correlation between the activation of the Ca^2+^ oscillations, enhanced expression of the genes encoding lipolysis proteins and diminution in the number of lipid droplets in the cytosol of adipocytes, in which Cx43 was activated for 60 min. This can be defined as a positive effect.

As shown in our experiments using Tirf-microscopy, ATP-containing vesicles were released by white adipocytes in response to the activation of Cx43. There is evidence that an increase in the level of expression of Cx43 and Panx1, which occurs during ischemia, enhances the ATP release into the extracellular space and thus ATP acts as an “alarm signal”. In adipose tissue, ATP secretions can perform different functions. For example, Chang and Cuatrecasas have shown that pre-exposure of white adipocytes to ATP inhibits glucose uptake by insulin [58]. Later studies convincingly prove that extracellular ATP causes an increase in cAMP levels and induces an increase in [Ca^2+^]_i_ through the activation of P2 purinoreceptors, which leads to the activation of protein kinase A, increased lipolysis and an a decrease in the leptin produced by white adipocytes [59,60]. ATP rapidly suppresses leptin secretion when insulin is added [61]. The analogue of ATP, BzATP, leads to a similar effect, which also enhances the lipolytic activity of adipocytes [60]. In addition, it is known that ATP is co-localized with norepinephrine in the sympathetic nerve terminals and released simultaneously in response to neuronal activity [62], acting as a co-transmitter for norepinephrine. Leptin production by white adipocytes was decreased in P2Y1 receptor knockout mice [63]. At the same time, with metabolic syndrome in humans, the expression level of P2 × 7 receptors in WAT significantly increases, the activation of which mediates the release of inflammatory cytokines [64]. Activation of these receptors under the action of ATP may contribute to the pathogenesis of the disease. On the other hand, high ATP concentrations are required for the activation of P2 × 7 receptors [65], which were unlikely to be realized under the conditions of our experiments.

At the level of intracellular signaling, it has been shown that, in addition to the mobilization of Ca^2+^ ions from intracellular stores, ATP can regulate the activity and voltage dependence of voltage-gated K^+^ currents in brown adipocytes [66], and may increase membrane conductance in single rat adipocytes [67].

Our previous experiments demonstrate that acetylcholine [68], norepinephrine [69,70] or calmodulin [71] may evoke short-term Ca^2+^ signals and simple or complex Ca^2+^ oscillations in white adipocytes, involving different mechanisms of Ca^2+^ spikes and oscillation generation [70]. Such differences in Ca^2+^ signaling should undoubtedly lead to different physiological effects in white adipocytes. Furthermore, in our previous studies, findings do not suggest that connexin channels contribute to generation and maintenance of Ca^2+^ oscillations regardless of the high expression of this protein in adipose tissue. However, there is evidence that Cx43 is involved in oscillatory Ca^2+^ responses of adipocytes during absorption of microparticles and other effects. Thus, Cx43 activation in adipose-derived stromal/stem cells has been found to be involved in regulation of Ca^2+^ oscillations that occur with involvement of NOS and internalization of quantum dots [71,72], while the role of these oscillations in regulation of differentiation or the development of these cells is yet to be understood.

The mechanisms of calcium oscillations can be divided into two large classes that depend on the receptor type, IP_3_R or RyR, used for mobilization of Ca^2+^ from the intracellular structures [73,74]. Our data show that in white adipocytes, Ca^2+^ oscillations activated by Cx43 in response to decreased extracellular Ca^2+^ occur exclusively due to activation of PLC and IP_3_R, despite the presence of functional ryanodine receptors in adipocytes [70]. At the same time, Ca^2+^ transients in response to addition of Ca^2+^-free solution were also suppressed by PLC, IP_3_R and G-protein inhibitors, but were not influenced by the inhibitors of connexin and pannexin hemichannels, probably due to other mechanisms of action. Nevertheless, these Ca^2+^ transients may also play an important role in regulation of lipolysis in adipocytes, as there is convincing evidence that activation of IP_3_Rs and PPARs promotes conversion of human white fat cells into beige adipocyte [75]. Cx43 expression can be regulated by renin and angiotensin II levels via activation of the extracellular signal-regulated kinase and NF-κB pathways [34], and these signaling systems are well designed in white adipose tissue, and closely linked to generation of Ca^2+^ oscillations and transients [76].

There is evidence of both the protective effect of connexin hemichannels and their negative effects in obesity. It has been shown that injections of the hemichannel inhibitor INI-0602 after 4 weeks of application leads to a decrease in the weight of high-calorie diet mice. It was found that inhibition of the gap junction hemichannel pathway affects the initial phases of nutritional disorders, i.e., prevents the initial stage of obesity development and does not affect the mice locomotor activity. However, in this in vivo study model, hemichannel inhibition was performed in brain cells, and the effects were observed in the body. At the same time, there is convincing evidence that connexins play a key role not only in adipogenesis but also in lipid metabolism of adipocytes [77]. It has been found that an increase in the expression level or activation of Cx43 in white adipose tissue is an effective approach against lipid accumulation in obesity and other metabolic diseases [78]. It has been shown that GJIC inhibition by 18 α-glycerrhetinic acid or adipocyte-specific Cx43 gene knockout reduced WAT beiging and found Cx43 to be important in maintaining mitochondrial integrity in brown fat [78,79], which is also observed in our studies, when the knockdown of Cx43 led to the accumulation of lipid droplets to the control level or even higher.

Furthermore, it has been shown that an increase in the [Ca^2+^]_i_ level after treatment of cells with capsaicin is due to activation of Cx43 and this causes lipolysis in the visceral depot of the WAT [77]. Cx43 inhibition in white adipocytes leads to activation of autophagy, enhancing ROS production; i.e., the activity of this connexin can play a protective role for adipose tissue against damage induced by oxidative stress [80]. The level of Cx43 expression is higher in BAT than in WAT [37] and Cx43 plays an important role during cold stress, by promoting the formation of WAT via cAMP transport between adipocytes [78]. In brown adipocytes, such as in white adipocytes, stimulation of the β3 adrenoreceptors increases ROS production, and inhibition of Cx43 enhances this process [81], which may cause oxidative stress.

In the regulation of lipolysis and lipogenesis of adipose cells, the following enzymes are crucial—hormone-sensitive lipase, sirtuins, insulin-like growth factor-2, triglyceride lipase, etc. [82]; so, in this work, we decided to investigate the expression of genes that encode these proteins during the generation of Ca^2+^ oscillations by white adipocytes through the activation of Cx43. A total of 24 h after the activation of Cx43 and Ca^2+^ oscillations in a calcium-free medium, an increase in the expression level of the *Lipe* gene encoding HSL was observed. In HSL^−/−^ mice, numerous morphological changes in white adipocytes were found, including heterogeneity of cell sizes, the emergence of a population of hypertrophied adipocytes and a large number of undifferentiated preadipocytes [83,84,85,86]. It is interesting that HSL^−/−^ mice do not show signs of obesity and to some extent are able to resist weight gain with a high-calorie diet [83,87] and are characterized by a decrease in circulating FFA in the blood, but accumulation of an increased amount of DAG in various tissues [83,85]. Consequently, the absence of HSL results in impaired white adipocytes differentiation or impaired mechanisms of accumulation of lipids in the form of DAG [86]. Lipolysis of the lipid inclusions accumulated by adipocytes occurs also due to activation of triglyceride lipase (ATGL) [88], and the expression level of the gene encoding it also significantly arises after activation of Cx43. In our experiments, Cx43 knockdown promoted the removal of the effect of connexin activation in response to the expression of *Lipe* and *Atgl* genes, which correlated with a rise in the number of lipid droplets in white adipocytes.

In addition to the lipases mentioned above, sirtuins also play important role in WAT physiology. Overexpression of Cx43 improves renal function in a db/db spontaneous diabetic model mice through increased Sirt1 expression, decreased HIF-1α expression and reduced extracellular matrix components. It was found that, in humans, obesity leads to a decrease in the level of Sirt1 in adipose tissue and its level is restored with a decrease in body weight [89]. Sirt1, through interaction with two PPARγ corepressors, the nuclear receptor corepressor (N-CoR) and the silencing mediator of the retinoid and thyroid hormone receptors (SMRT), can suppress adipogenesis [90]. Overexpression of Sirt1 inhibits adipogenesis in the 3T3-L1 cells [91,92], as well as determines the differentiation of mesenchymal stem cells into myogenic cells, but not into preadipocytes, through interaction with the Wnt signaling pathway [93]. In our experiments, there is an increase in Sirt1 expression upon activation of Cx43, which, on the one hand, can prevent the accumulation of lipid inclusions, which was recorded using OilRed, and, on the other hand, can promote adipocyte differentiation. Sirt1 activation also promotes the phosphorylation of AMPK and inhibits the synthesis of fatty acids and an increase in the number of lipid inclusions in adipose cells in response to high glucose levels [94]. In our experiments, suppression of the *Sirt1* gene expression after application of the Ca^2+^-free medium with CBX or to Cx43-KD adipocytes can abolish the anti-lipolytic effect of Cx43 activation through this signaling pathway.

Sirt3 is required for the activation of the bioenergetic functions of mitochondria in the early stages of adipocyte differentiation. Silencing of Sirt3 decreases the protein level of the forkhead box O3a (FoxO3a) transcription factor and subsequently downregulates the expression of several antioxidant enzymes and increases oxidative stress in mesenchymal stem cells after adipogenic induction. Therefore, Sirt3 depletion reduces the ability of mesenchymal stem cells to undergo adipogenic differentiation and leads to adipocyte dysfunction [95].

Another important factor, insulin-like growth factor-2 (IGF2), is a growth-promoting polypeptide that has a high degree of structural homology with insulin, a widely expressed peptide hormone in cell division [96]. IGF2 is a key factor regulating cell proliferation, growth, migration, differentiation, survival and lipid metabolism [97]. IGF2 is synthesized mainly by the liver, but it is also produced locally by many tissues, where it acts in an autocrine or paracrine manner [98].

It is known that the level of IGF2 protein in the blood serum is considerably higher in patients with metabolic syndrome, and the level of expression of the gene encoding IGF2 is also increased in obese mice on a high-calorie diet [99]. IGF2 promotes the proliferation and differentiation of preadipocytes line 3T3-L1 and enhances accumulation of lipid inclusions by these cells [100]. Overexpression of IGF2 contributes to formation and accumulation of lipid inclusions by hepatocytes in adult mice [101,102]. Interestingly, in our experiments, the activation of Cx43 after application of Ca^2+^-free medium resulted in suppression of the expression of *Igf2* gene encoding insulin-like growth factor-2 (IGF2), which can promote lipolysis. It is known that insulin markedly increases the rate of synthesis and accumulation of triglyceride by 3T3-L1 adipocytes [100]. At the same time, the use of a Ca^2+^-free medium in combination with CBX contributed to a significant increase in the expression of *Igf2*, as well as the knockdown of Cx43, which correlated with the inhibition of lipolysis and the appearance of a large number of lipid droplets.

There is evidence that in type 2 diabetes there is an increased level of [Ca^2+^]_i_ in endothelial cells [103], and a decrease in calcium intake leads to an increase in body weight and adipose tissue due to the paradox of an increase in the level of [Ca^2+^]_i_. There is a hypothesis that in response to a decrease in dietary calcium intake, an increase in the level of parathyroid hormone and 1,25-dihydroxyvitamin D (calcitriol) occurs, which leads to an increase in [Ca^2+^]_i_ and activates the accumulation of triglycerides by adipocytes [15,16]. Drastic alterations in Ca^2+^ ions can regulate numerous physiological processes, ranging from the normal functioning of tissues to the induction of metabolic disorders. Recently, Cx43 mimetics have been of great interest in the treatment of metabolic disorders [104,105]. Our study contributes to understanding the signaling mechanisms of regulation of lipolysis by Ca^2+^ ions.

## 4. Materials and Methods

Experimental protocols were approved by the Bioethics Committee of the Institute of Cell Biophysics. Experiments were carried out according to Act708n (23 August 2010) of the Russian Federation National Ministry of Public Health, which states the rules of laboratory practice for the care and use of laboratory animals, and the Council Directive 2010/63 EU of the European Parliament on the protection of animals used for scientific purposes.

### 4.1. Isolation of Primary White Preadipocytes

Cell cultures were prepared as described in detail previously [106]. All studies were approved by the Animal Ethics Committee of the Institute of Cell Biophysics. NMRI mice (aged 3–5 weeks) were decapitated after a brief (45–60 s) anesthesia with carbon dioxide before sacrifice. Mice were subjected to cervical dislocation and disinfected with 70% ethanol prior to dissection. All operations were performed in a sterile environment on ice. White adipose tissue was removed from the epididymal fat depot and placed in a Petri dish with cold DMEM medium. Scissor-minced white adipose tissue was transferred into a tube containing sterile DMEM with 7 mg type II collagenase (Sigma-Aldrich, USA) and 4% bovine serum albumin (BSA, free from fatty acids). Then the tissue was incubated for 18 min at 37 °C. To stop the enzymatic reaction, the tube was chilled on ice for 20 min with intermittent shaking followed by filtration through a 250 µm filter and centrifugation at 1000× *g* for 10 min. The pellet was then resuspended in cold DMEM medium, filtered through 50 µm filters and centrifuged at 1000× *g* for 10 min. Finally, the pellet was resuspended in cultural medium containing DMEM (Sigma-Aldrich, USA), 10% fetal bovine serum (FBS; Gibco, USA), 4 mM L-glutamine, 5 nM insulin [107,108], 0.004% gentamicin and 25 µg/mL sodium ascorbate (Sigma-Aldrich, USA). The obtained suspension contained preadipocytes, since mature adipocytes carry vesicles of fat and do not precipitate under the given conditions.

### 4.2. Cultures of Primary White Preadipocytes

A total of 100 µL of culture medium containing 3 × 10^4^ preadipocytes were placed on round cover glasses (25 mm in diameter), which were then transferred into 35 mm Petri dishes. Six hours after adhesion of the cells to the glass, additional culture medium was added to the Petri dishes. On the third day the medium in the dishes was replaced with a fresh portion of medium, which included 10 nM cytosine arabinoside (Sigma-Aldrich, USA) to suppress the proliferation of fibroblasts, and incubation in a CO_2_ atmosphere was continued for 8 h. After that the medium was replaced with fresh culture medium. White pre-adipocytes in culture actively proliferate within 5 days. Upon reaching confluence, cells begin to spontaneously differentiate and accumulate lipid droplets. On the ninth day of culture, the cells form a monolayer and become differentiated.

### 4.3. Transfection with Small Interfering RNA (siRNA)

When the cell confluence reached at 40% (5 days in vitro), cells were transfected with siRNA against mouse Gja1 (Cx43) (siRNA ID 158724, Thermo Fisher Scientific, USA) and Panx1 (siRNA ID 185206, Thermo Fisher Scientific, USA) using lipofectamine RNAiMax (Invitrogen, USA) according to the manufacturer’s instructions. Cultures transfected with an siRNA whose sequence differed from that of the siRNA against Cx43 or Panx1 (Scrambled) were used as controls. After incubating the white fat cells with siRNA-reagent mixtures in Opti-MEM (Gibco, USA) containing 50 pM of siRNA, Gja1 were added for 6 h. Then the cultural medium was changed, and the cells were incubated for an additional 48 h. The efficiency of Gja1 knockdown was assessed using RT-qPCR and immunocytochemical staining of cells with antibodies against Cx43. The efficiency of the knockdown was at least 85–90%. Experiments were performed on 9 DIV.

### 4.4. Immunocytochemical Staining of Adipocytes

Cx43 in cells was detected using the immunocytochemical staining method. The cells were fixed with 4% paraformaldehyde solution in PBS for 20 min. This was followed by three 5-min washing of cells with ice-cold PBS. For permeabilization, cells were incubated for 15 min in 0.1% Triton X-100 solution. Blocking of nonspecific binding of antibodies was performed using a 10% solution of donkey serum in PBS. The cells were incubated in blocking solution for 30 min at room temperature. The primary antibodies were loaded for 12 h at 4 °C. Connexin 43 monoclonal antibody (CX-1B1, Thermo Fisher Scientific, USA, Cat# 13-8300), dissolved in 1% donkey serum in a ratio of 1:500, were used as primary antibodies. After incubation with primary antibodies, the cells were washed three times with PBS with an interval of 5 min. Then the cells were loaded with secondary antibodies, which were donkey polyclonal secondary antibody to mouse IgG—H&L (Alexa Fluor-594, Abcam, RRID: AB_2732073). Incubation with secondary antibodies was performed at room temperature in the dark for 90 min. Secondary antibodies were dissolved in PBS at a ratio of 1:200. Antibody fluorescence was visualized using an inverted laser scanning confocal microscope (Leica TCS SP5, Leica, Germany). An argon laser with a 488 nm band was used to excite the fluorescence. Emissions were recorded in the range 505–565 nm. Cell nuclei were stained with a Draq5 probe.

### 4.5. The Measurement of Cytosolic Calcium Concentration

The measurement of the cytosolic [Ca^2+^] was performed by fluorescent microscopy using Fura-2AM (Molecular probes, USA), a ratiometric fluorescent calcium indicator. Cells were loaded with the probe dissolved in Hanks balanced salt solution (HBSS) composed of (mM) 156 NaCl, 3 KCl, 2 MgSO_4_, 1.25 KH_2_PO_4_, 2 CaCl_2_, 10 glucose, containing 10 mM HEPES, pH 7.4, at a final concentration of 5 µM at 37 °C for 40 min with a subsequent 15 min washout. The coverslip containing the cells loaded with Fura-2 was then mounted in the experimental chamber. During the experiment we used a perfusion system, which enables complete replacement of the cell bathing solution within 30 s. We used an Axiovert 200M-based imaging system (Carl Zeiss, Germany) equipped with an HBO100 mercury lamp, AxioCam HSm CCD camera and MAC5000 high-speed excitation filter wheel. Fura-2 fluorescence was excited at two wavelengths using band-pass filters BP 340/30 and BP 387/15; fluorescence was registered in the wavelength range of 465–555 nm. Excitation light intensity was lowered using 25 and 5% neutral density filters in order to prevent phototoxicity. Image frames were acquired at 3 s intervals with a Plan Neofluar 10×/0.3 objective. The time lapse image sequences were analyzed using ImageJ 1.44 (NIH Image, Bethesda, MD, USA). Graphs were plotted using OriginPro 8.0 software Microcal Software Inc., Northampton, MA, USA). Statistical analysis was performed using the same software. Results are presented as the means ± standard error (SE) or as the representative calcium signal of the cells.

### 4.6. Assessment of Hemichannel Open Probability by Dye Loading

Connexin hemichannels are permeable to the fluorescent dye carboxyfluorescein (376 Da) and in an open state can act as conduits of carboxyfluorescein transport across the membrane in accord with the concentration gradient of the dye [45,109]. Carboxyfluorescein (100 µM) was added to the cell incubation media for 10 min, resulting in background connexin-mediated dye loading, followed by application of the experimental stimulus. Then the cells were washed for 5 min, and the degree of intracellular carboxyfluorescein accumulation (dye loading) was assessed by measuring the intensity of carboxyfluorescein fluorescence in individual cells. Images of carboxyfluorescein fluorescence were taken using an inverted confocal microscope (Leica TCS SP5, Leica, Germany) before and after addition of the dye, after the application of the Ca^2+^-free stimulus in the presence of the dye in the media and after the washout of carboxyfluorescein. Using ImageJ software, regions of interest were drawn around the cell bodies of white adipocytes and the mean pixel intensity for all the cells in the field of view was calculated. Background fluorescence was subtracted.

### 4.7. Quinacrine Staining

To visualize the ATP-containing vesicles, adipocytes were stained with 5 μM quinacrine in Hanks solution containing 10 mM HEPES at 37 °C for 15 min. Quinacrine, a derivative of acridine, is a weak base that binds ATP with high affinity [47,110]. After the incubation the cells were washed 5 times with Hanks solution and used to visualize the vesicles using TIRF microscopy.

### 4.8. Total Internal Reflection Fluorescence (TIRF) Microscopy

To visualize and investigate the dynamics of the release from adipocytes of ATP-containing vesicles stained with quinacrine, TIRF microscopy was used. An inverted TIRF microscope (IX71, Olympus, Japan) equipped with an immersion oil lens with a high numerical aperture (60 ×/1.65 NA) and a cooled high-resolution camera (Hamamatsu, Japan) was used for this. Series of images were obtained and analyzed using the Olympus Cell software (Olympus). The quinacrine fluorescence was excited using an argon laser at a wavelength of 488 nm, emission was recorded at a wavelength range 500–530 nm. To assess changes in the fluorescence intensity, an area of interest (ROI) was selected and fluorescent granules containing ATP were detected. A decrease in the fluorescence intensity in the region of interest testified to the secretion of vesicles into the extracellular space. Experiments were performed at 37 °C.

### 4.9. Total RNA Isolation

Total RNA was isolated from the primary culture of white adipocytes using the Mag Jet RNA reagent kit (Thermo Scientific, USA) according to the manufacturer’s instructions. The quality of the RNA was assessed by electrophoresis in 2% agarose gel in TBE buffer in the presence of ethidium bromide (1 μg/mL). The RNA concentration was measured using a NanoDrop 1000 c spectrophotometer (Thermo Scientific, USA). cDNA was synthesized using the RevertAid H Minus First Strand kit according to the protocol recommended by the manufacturer (Thermo Scientific). Single-stranded cDNA preparations were used as a template for real-time PCR analysis.

### 4.10. Real-Time Polymerase Chain Reaction (RT-qPCR)

Each PCR was performed in a 25 μL mixture composed of 5 μL of qPCRmix-HS SYBR (Evrogen, Moscow, Russia), 1 μL (0.2 μM) of the primer solution, 17 μL water (RNase-free) and 1 μL cDNA. The Dtlite Real-Time PCR System (DNA-technology, Moscow, Russia) was used for amplification. The amplification process consisted of the initial 5 min denaturation at 95 °C, 40 cycles of 30 s denaturation at 95 °C, 20 s annealing at 60–62 °C and 20 s extension step at 72 °C. The final extension was performed for 10 min at 72 °C. The sequences of the used primers are presented in Table 1. All the sequences were designed with FAST PCR 5.4 and NCBI Primer-BLAST software. The data were analyzed with Dtlite software (DNA-technology, Moscow, Russia). The expression of the studied genes was normalized to the gene encoding glyceraldehyde 3-phosphate dehydrogenase (GAPDH). Data were analyzed using Livak’s method [111].

### 4.11. Western Blot

Cells were homogenized with Cell Lysis Buffer containing 100 mM Tris–HCl, pH 8.0, 0.15 mM NaCl, 1 mM EDTA and 1 mM phenylmethane sulfonate fluoride (PMSF). The lysates were cleared by centrifugation at 3000× *g* for 5 min at 4 °C. Cell extracts contained 30 μg of total protein. Proteins were separated by SDS–PAGE on 10% or 12% polyacrylamide gels and were transferred onto a nitrocellulose membrane (Thermo Scientific). Membranes were blocked for 2 h at room temperature in 5% non-fat dry milk in PBS. Nitrocellulose blots were subsequently incubated overnight at 4 °C with primary antibodies (all 1:5000). Thereafter, blots were incubated for 2 h with the secondary antibody conjugated to horseradish peroxidase (1:5000, ab205719). Immunoreactive bands were visualized by detection of peroxidase activity by chemiluminescence with Western ECL reagents (SuperSignal West Pico PLUS Chemiluminescent Substrate, Thermo Scientific). We used HSL polyclonal antibody (LifeSpan BioSciences, USA, LS-C417602), ATGL monoclonal antibody (ThermoFisher, USA, MA5-14990) and Anti-GAPDH antibody (Invitrogen, USA, PA1-16777). Protein expression was quantified using ImageJ software.

### 4.12. Lipid Droplets Assessment

Adipogenic differentiation of the cells was histologically assessed by staining with Oil Red. Samples were fixed with 3.7% buffered formalin for 1 h. Then the cells were washed from the formalin solution. Then the cells were stained with Oil Red solution (3 mg/mL Oil Red in 60% isopropanol; Sigma-Aldrich, USA) for 2 h and washed three times with PBS. Then the samples were dried in a thermostat at 37 °C. Samples were imaged using an Axiovert 200 M based imaging system (Carl Zeiss, Germany). ImageJ software was used in order to analyze images.

### 4.13. Free Glycerol Release Assay

To measure the free glycerol concentrations, we used the Free Glycerol Assay kit (Gibco, USA, ab65337). The cells were grown up to 8 days in vitro, then the medium was replaced with a Ca^2+^-free one for 60 min, after which the cultures were returned to the CO_2_ incubator for 24 h. After 24 h, the cells were washed three times with PBS, the cells were removed from the dishes and lysed. Further manipulations were performed in accordance with the instructions of the developer. Fluorescence registration (Ex/Em = 535/587 nm) was performed using a Spark10M multifunctional plate reader (Tecan, Männedorf, Switzerland).

### 4.14. Statistical Analysis

All presented data were obtained from at least three cell cultures from 2–3 different passages (*n*—number of the experiments). All values are given as the mean ± standard error (SE). The differences between the columns were estimated with a paired *t*-test. Two-way or one-way analysis of variance (ANOVA) followed by the post-hoc Tukey–Kramer test was used for multiple group comparisons. The statistical tests were performed with GraphPad Prism 5 software.

## 5. Conclusions

Thus, in differentiated white adipocytes, the reduced concentration of [Ca^2+^]_i_ led to the generation of two types of Ca^2+^ signals, such as Ca^2+^ oscillations and Ca^2+^ transients. Ca^2+^ oscillations occurred due to the activation of the Cx43 hemichannels and vesicular ATP secretion, leading to paracrine activation of most white adipocytes in vitro. The mechanism of Ca^2+^ oscillation generation, apparently, is based on the mobilization of Ca^2+^ ions from the thapsigargin-sensitive endoplasmic reticulum pool via IP_3_R, and is switched on by the activation of P2Y1 purinoreceptors and G-proteins. Long-term Ca^2+^ oscillations in adipocytes influence the expression level of the genes involved in the regulation of lipogenesis/lipolysis and altered the balance of both processes in favor of activation of lipolysis, resulting in a diminished lipid droplets number.

## Figures and Tables

**Table 1:** Primer sequences for real-time polymerase chain reaction (RT-PCR).

NM_001289726 GAPDH	Forward 5′-tccactcacggcaaattcaac-3′ Reverse 5′-cggcatcgaaggtggaagag-3′
NM_010719 Lipe	Forward 5′-gagcactacaaacgcaacgagaca-3′ Reverse 5′-aaattcagccccacgcaactct-3′
NM_019812 Sirt1	Forward 5′-ctttcagaaccaccaaagcgga-3′ Reverse 5′-acagaaaccccagctccagtca-3′
NM_022433 Sirt3	Forward 5′-acctttgtaacagctacatgcacggt-3′ Reverse 5′-ccatcacatcagcccatatgtcttc-3′
NM_010514 Igf2	Forward 5′-cctcctggagacatactgtgccac-3′ Reverse 5′-tgtctccaggtgtcatattggaagaa-3′
NM_010288 Gja1	Forward 5′-cttcaatggctgctcctcacca-3′ Reverse 5′- gctcgctggcttgcttgttgt-3′
NM_025802 Atgl	Forward 5′-tcattcctcctaccctccaag-3′ Reverse 5′-atcgaagtccatctctgtagc-3′

## Data Availability

Data will be made available on reasonable request.
